# Anti-SARS-CoV-2 Titers Predict the Severity of COVID-19

**DOI:** 10.3390/v14051089

**Published:** 2022-05-18

**Authors:** Antonios Kritikos, Sophie Gabellon, Jean-Luc Pagani, Matteo Monti, Pierre-Yves Bochud, Oriol Manuel, Alix Coste, Gilbert Greub, Matthieu Perreau, Giuseppe Pantaleo, Antony Croxatto, Frederic Lamoth

**Affiliations:** 1 Institute of Microbiology, Lausanne University Hospital, University of Lausanne, 1011 Lausanne, Switzerland; alix.coste@chuv.ch (A.C.); gilbert.greub@chuv.ch (G.G.); antony.croxatto@ne.ch (A.C.); frederic.lamoth@chuv.ch (F.L.); 2 Infectious Diseases Service, Lausanne University Hospital, University of Lausanne, 1011 Lausanne, Switzerland; sophie.gabellon@unil.ch (S.G.); pierre-yves.bochud@chuv.ch (P.-Y.B.); oriol.manuel@chuv.ch (O.M.); 3 Intensive Care Unit, Lausanne University Hospital, University of Lausanne, 1011 Lausanne, Switzerland; jean-luc.pagani@chuv.ch; 4 Service of Internal Medicine, Lausanne University Hospital, University of Lausanne, 1011 Lausanne, Switzerland; matteo.monti@chuv.ch; 5 Service of Immunology and Allergy, Lausanne University Hospital, University of Lausanne, 1011 Lausanne, Switzerland; matthieu.perreau@chuv.ch (M.P.); giuseppe.pantaleo@chuv.ch (G.P.); 6 ADMED Microbiology, 2300 La Chaux-de-Fonds, Switzerland

**Keywords:** serology, antibody response, SARS-CoV-2, COVID-19, outcome, disease severity

## Abstract

Coronavirus disease 2019 (COVID-19) due to SARS-CoV-2 is associated with a wide spectrum of disease, ranging from asymptomatic infection to acute respiratory distress syndrome. Some biomarkers may predict disease severity. Among them, the anti-SARS-CoV-2 antibody response has been related to severe disease. The aim of this study was to assess the correlation between the anti-SARS-CoV-2 serological response and COVID-19 outcome. Demographic, clinical, and biological data from nasopharyngeal-PCR confirmed COVID-19 hospitalized patients were prospectively collected between April and August 2020 at our institution. All patients had serial weekly serology testing for a maximum of three blood samples or until discharge. Two different serological assays were used: a chemiluminescent assay and an in-house developed Luminex immunoassay. Kinetics of the serological response and correlation between the antibody titers and outcome were assessed. Among the 70 patients enrolled in the study, 22 required invasive ventilation, 29 required non-invasive ventilation or oxygen supplementation, and 19 did not require any oxygen supplementation. Median duration of symptoms upon admission for the three groups were 13, 8, and 9 days, respectively. Antibody titers gradually increased for up to 3 weeks since the onset of symptoms for patients requiring oxygen supplementation with significantly higher antibody titers for patients requiring invasive ventilation. Antibody titers on admission were also significantly higher in severely ill patients and serology performed well in predicting the necessity of invasive ventilation (AUC: 0.79, 95% CI: 0.67–0.9). Serology testing at admission may be a good indicator to identify severe COVID-19 patients who will require invasive mechanical ventilation.

## 1. Introduction

Coronavirus disease 2019 (COVID-19) due to SARS-CoV-2 is associated with a wide spectrum of symptoms, ranging from asymptomatic infection to acute respiratory distress syndrome [1,2,3,4]. COVID-19 is often biphasic, with acute respiratory distress syndrome developing after a few days of initially mild symptoms (usually above one week) in some patients as a consequence of pro-inflammatory cytokine release [5]. This detrimental role of the immune response has led to the hypothesis that the antibody-mediated immune response could contribute to the disease severity [6,7,8].

Early assessment of the disease severity and prediction of outcome might help physicians to optimize the patient’s medical care and resource management [9,10,11]. Some prediction tools have been developed based on epidemiologic, clinical, or laboratory features [12] but their performances have been limited and most of them have not been prospectively evaluated [13]. The importance of the anti-SARS-CoV-2 antibody response has been suggested as an indicator of the degree of disease severity. However, reports that relate antibody titers to disease severity showed discordant results [5,14,15,16,17,18,19,20,21,22,23,24,25,26,27,28,29].

In order to evaluate the kinetics of the anti-SARS-CoV-2 humoral immune response and its role as a prognostic factor, we conducted a prospective observational study in hospitalized patients with serial monitoring of their immunoglobulin G (IgG) response to SARS-CoV-2 measured by two different methods during the course of infection. Our aim was to assess the role of serology as a pragmatic stratification tool to evaluate the patient’s prognosis upon hospital admission.

## 2. Materials and Methods

### 2.1. Study Design and Participants

This was a prospective observational study conducted at Lausanne University Hospital (CHUV) between 1 April 2020 and 1 August 2020. All hospitalized patients with acute respiratory symptoms and COVID-19 infection confirmed by a positive nucleic acid amplification test (NAAT) for SARS-CoV-2 in a nasopharyngeal swab or any other respiratory specimen were included if the hospital stay was anticipated to last for more than 48 h and provided that informed consent was obtained. Exclusion criteria were: (1) nosocomial acquisition of COVID-19 and (2) patients with documented concomitant active infection at the time of COVID-19 diagnosis.

Patients were classified according to the severity of COVID-19 considering the entire hospital stay (i.e., highest degree of severity) using a modified ordinal scale as recommended by the World Health Organization research and development (WHO R&D) Blueprint group [30,31] as follows: (1) No oxygen therapy; (2) oxygen by masks or nasal prongs; (3) non-invasive ventilation or high-flow oxygen; (4) mechanical ventilation; (5) mechanical ventilation and additional organ support (vasopressors, renal replacement therapy or extracorporeal membrane oxygenation); and (6) death. Included patients were subsequently divided into three groups: mild cases (category 1), moderate cases (categories 2 and 3), and severe cases (categories 4 to 6). Demographic, clinical, and laboratory data were collected from the electronic health records from our hospital. The timing of COVID-19 was assessed from the day of the start of first symptoms (i.e., fever or any flu-like or respiratory symptoms) as reported by the patient or their relatives. During the study period, patients were managed according to the internal guidelines, issued by a multidisciplinary consensus of various specialists.

### 2.2. Serological Testing

Serial sera were collected from participants at the day of inclusion (=day 0), and then at days 7 and 14 provided that they were still hospitalized after two weeks, otherwise until discharge. The serum samples were tested by two serological methods for the anti-SARS-CoV-2 IgG antibody detection: the ChemiLuminescent ImmunoAssay (CLIA) LIAISON^®^XL SARS-CoV-2 IgG Kit (DiaSorin, Saluggia, Italy, hereafter referred as CLIA) and an in-house Luminex ImmunoAssay (hereafter coined Luminex), as previously described [32]. SARS-CoV-2 specific antibody targets were the S1 monomer and N protein for CLIA and the trimeric S protein including the receptor binding antigen for Luminex [32]. Results were expressed as the arbitrary units per milliliter (UA/mL) for CLIA (cut-off of positivity: >14 UA/mL) and as the median fluorescent intensity (MFI) of the sample/negative control ratio for Luminex (cut-off of positivity: >6).

### 2.3. Statistical Analysis

Descriptive statistics were presented as numbers (percentages) for the categorical variables and as the mean ± standard deviation (SD) for the normally distributed continuous variables or as the median [interquartile range (IQR)] for continuous variables with a skewed distribution. The Chi-square or Fisher’s exact test were used for the categorical variables and the Wilcoxon matched-pairs rank test or one-way ANOVA for continuous variables where appropriate. Sensitivity and 95% confidence interval (CI) were calculated to assess the diagnostic performances of the two serological assays used in the study. All statistical analyses were performed using GraphPad Prism version 8.3.0 for Windows (GraphPad Software, San Diego, CA, USA).

### 2.4. Ethics

This project was conducted in accordance with the Declaration of Helsinki, the principles of Good Clinical Practice, and the Swiss Human Research Act (HRO). The project received approval from the Ethics Committee of Canton Vaud, Switzerland (2020–00697). All patients or their legal representatives (in case of inability of the patient) provided written informed consent for the serum sampling and collection of clinical data.

## 3. Results

### 3.1. Patient Characteristics

Seventy COVID-19 patients were enrolled in the study, of which 22 (31%), 29 (41%), and 19 (27%) were classified as severe, moderate, and mild disease, respectively. Eleven (50%) patients of the severe group were admitted directly to the ICU. Patients were predominantly males (n = 40) with a median age of 60 years-old (IQR 51–73). The baseline underlying conditions of the patients and characteristics of infection are presented in Table 1 and Table 2, respectively. Obesity and dyslipidemia were more frequently associated with moderate disease, while cough, dyspnea, and radiological infiltrate were more commonly observed in subjects with severe disease than in those with moderate and mild disease. D-dimer and CRP were both significantly higher in subjects with severe disease. The median duration of symptoms upon admission for the three groups was 13 (IQR: 9–15), 8 (7–12), and 9 (3–11) days, respectively (*p* = 0.55). Forty-four (63%) patients received at least one anti-SARS-CoV-2 treatment during their hospital stay (Table S1).

### 3.2. Diagnostic Performances and Kinetics of Serological Assays

A total of 133 sera from 70 patients were analyzed with both the Luminex and CLIA assays (median 2 sera per patient, range 1–3). The proportion of patients with a positive serology testing for Luminex/CLIA at D0, D7, and D14 from hospital admission was 84/63%, 97/97%, and 100/97%, respectively.

**Figure 1:**
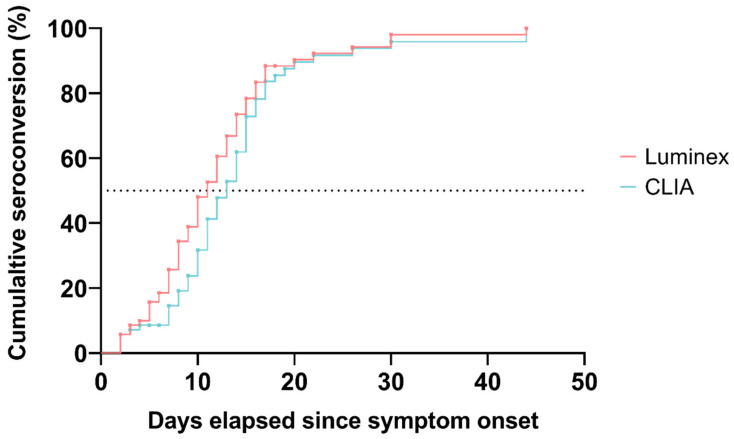
The cumulative proportion of patients who seroconverted according to both serological methods: Luminex (red), CLIA (blue). The dotted line represents the 50% cumulative seroconversion threshold.

Diagnostic performance was calculated for different time-points from the onset of symptoms. The cumulative IgG seroconversion rate for Luminex/CLIA was 25/14%, 73/62%, and 98/96% at days 7, 14, and 28, respectively (Figure 1). The median time from the onset of symptoms to IgG seroconversion was 11 and 13 days for Luminex and CLIA, respectively.

**Figure 2:**
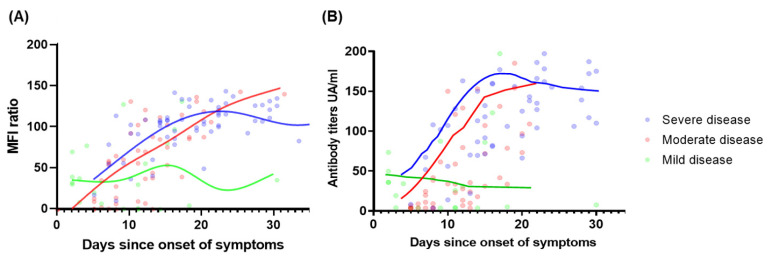
Trends in antibody evolution stratified by disease severity. Smooth fit curves for antibody kinetics tested with (**A**) Luminex and (**B**) CLIA.

The evolution of IgG antibody titers measured by both the Luminex and CLIA differed significantly according to the severity of disease, as illustrated in Figure 2. While IgG titers in mild disease remained low and stable, an increase in IgG titers was observed until day 14 after symptoms onset with CLIA (day 18 with Luminex), followed by sustained high antibody levels among patients with moderate/severe disease.

### 3.3. Correlation of Baseline Antibody Levels with the Severity of Disease

**Figure 3:**
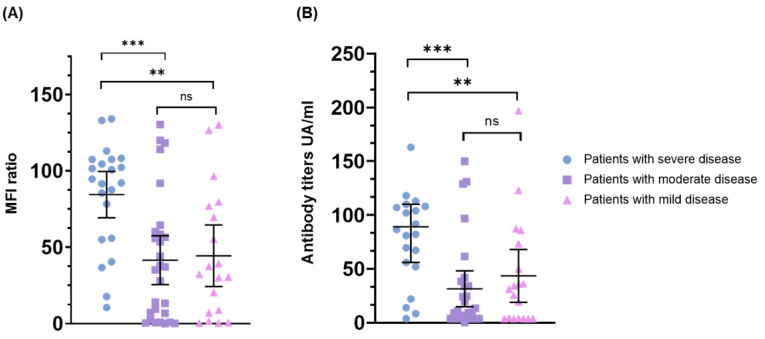
The antibody titers at admission according to clinical stratification. Panel (**A**) Luminex assay, panel (**B**) CLIA. Scatter plot with lines at the median and whiskers showing the 95% confidence interval (CI). *** = *p* < 0.001, ** = *p* < 0.01, ns = non-significant, MFI = median fluorescence intensity.

The IgG antibody titers at admission (=day 0) were compared between the three groups of patients (mild, moderate and severe disease) in order to assess the role of the baseline quantitative serology in predicting the disease severity and outcome. A significant difference in IgG levels was observed between the severe cases versus the moderate and mild cases (*p* < 0.01) (Figure 3).

**Figure 4:**
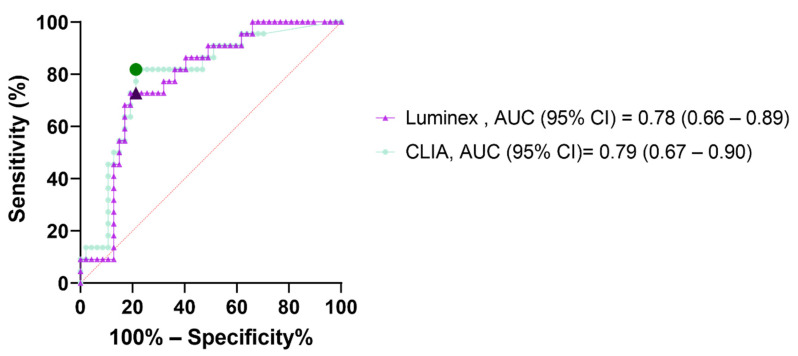
The receiver operating curves (ROC curves) for the performance of the serology testing to predict the necessity for invasive ventilation (AUC = area under the ROC curve, 95% CI = 95% confidence interval). The marked symbols show the performances of the optimal cut-off values.

The receiver operating curves (ROC) of the initial serology testing (D0 = admission) to predict severe disease is shown in Figure 4. The performances of a single determination of IgG upon admission were comparable for the Luminex and CLIA with areas under the ROC curves (AUC) of 0.78 (95% CI: 0.66–0.89) and 0.79 (0.67–0.90), respectively. The optimal cut-off points to predict the need for mechanical ventilation were defined based on the ROC curves and their diagnostic performances are shown in Table 3. These were compared with the diagnostic performances of other laboratory markers that exhibited a significant difference (*p* < 0.05) between the three groups of COVID-19 severity grading in Table 1: CRP (*p* < 0.01) and D-dimer (*p* = 0.03).

The optimal performance was obtained for a CLIA antibody titer at admission above 50.8 UA/mL with sensitivity, specificity, positive predictive value (PPV), and negative predictive values (NPVs) of 82% (95% CI: 61–93), 79% (65–88), 63% (49–76), and 91% (80–96), respectively.

The Luminex assay also proved to be able to predict the necessity for invasive ventilation (Figure 4 and Table 3).

Based on the ROC curves (data not shown), the optimal performances for the D-dimer and CRP were obtained at a cut-off of >1000 ng/mL and >100 mg/L, respectively. Sensitivity, specificity, PPV, and NPV were inferior to that of the CLIA IgG titers (>50.8 UA/mL) and the Luminex IgG titers (>77.6 MFI ratio), as shown in Table 3.

Among the patients with a CLIA IgG titer >50.8 UA/mL at admission (n = 28), 8/28 were already intubated or intubated immediately at admission, and 10/28 required mechanical ventilation later during their hospital stay (median 2, range 1–16 days after admission). The remaining 10 patients did not require mechanical ventilation (five were mildly and five moderately ill).

In order to account for a possible bias confounded by higher antibody levels seen later in the course of the disease, we analyzed the antibody titers in the subgroups of patients presenting with the same duration of symptoms. Higher antibody levels correlated with the severity of the disease with a more pronounced significant effect for patients with 11–15 days of symptom duration compared to 5–10 days (Figure S1).

## 4. Discussion

The role of SARS-CoV-2 serological testing for the diagnosis of COVID-19 is currently limited, aside from seroprevalence studies, to specific situations such as in the case of late immune complications of the disease (e.g., multisystem inflammatory syndrome in children or meningoencephalitis) [33,34,35,36,37]. While nucleic acid amplification tests (NAAT) remain the gold standard for COVID-19 diagnosis [35,38], their sensitivity decreases beyond the first week of symptoms, while serology achieves a >90% sensitivity beyond the third week [32,39,40]. While patients with complicated COVID-19 usually require hospitalization beyond one week of symptom onset (i.e., median 10 days in the present study), serological testing may be combined to NAAT at hospital admission to increase the diagnostic performance for patients who have >10 days of symptoms and have not been tested during the early phase of the disease. Indeed, we observed that a majority of patients already had a positive SARS-CoV-2 serology at admission, particularly with the Luminex assay (84%), which was associated with earlier documented seroconversion during the first two weeks of symptoms compared to the CLIA. The higher sensitivity of the Luminex assay might reflect the larger number of antigenic epitopes targeted compared to the CLIA methods used [32].

The main objective of this work was to prospectively assess the kinetics of SARS-CoV-2 serological titers and to correlate these profiles with the severity and course of infection.

Previous publications have reported conflicting results regarding the correlation of antibody titers and the outcome of SARS-CoV-2 infection. Many studies have reported that more severe cases have higher IgG titers [5,14,29,41], but others failed to detect any difference in IgG titers among the mild and severe cases [25,26,27]. High IgG titers could lead to alveolar transudation and the formation of immune complexes with viral particles and as such, activate the complement system and trigger a more pronounced inflammatory response [42]. It is worth mentioning though, that definitions of the disease’s severity vary somewhat among the different studies.

In the present study, we observed two distinct patterns of serological response over time: elevated and sustained antibody titers in patients with moderate/severe COVID-19 and a low and/or transient response in mild cases. The IgG levels were significantly higher at admission among patients with severe disease requiring mechanical ventilation compared to those with mild/moderate disease. Therefore, we tested the hypothesis of a possible role of SARS-CoV-2 serology as a stratification tool to predict the need for mechanical ventilation at hospital admission. The best performance was obtained using the commercialized CLIA kit with a positive predictive value of 63% and a negative predictive value of 91% for the cut-off of 50.8 UA/mL. It is noteworthy that about one-third of these patients with initial IgG titers >50.8 UA/mL already presented with the criteria of severe disease at admission with immediate transfer to the ICU and start of mechanical ventilation. Therefore, the utility of such an approach might be restricted to the remaining cases among which the risk of subsequent ICU admission and mechanical ventilation is high. Identification of such high risk patients is particularly important, as these patients may benefit from early interventions such as the administration of immunomodulators (tocilizumab) [43] or monoclonal antibodies, which have demonstrated some efficacy if not delayed [44,45].

Some previous studies have highlighted the role of other biomarkers such as CRP, D-dimer, ferritin, or lymphocyte count for the discrimination between mild and severe COVID-19 [12,46,47]. In the present study, we observed a significant difference in CRP and D-dimer levels at admission between the three severity grading groups, but not for the ferritin levels and lymphocyte count. Interestingly, serology testing for IgG at admission performed better than CRP and D-dimer for the identification of patients with or at risk of severe disease.

A limitation of the present study consists of the small number of recruited patients and the relative short-time frame of the study. The study was conducted during the first COVID-19 wave and was interrupted in the summer of 2020 once the number of hospitalized COVID-19 patients had drastically declined. Therefore, despite the limited number of cases, this dataset represents a very homogenous population, notably regarding the circulating SARS-CoV-2 variant and the therapeutic approach. Whether these results would be reproducible with other variants and/or among vaccinated individuals is unknown and would deserve further investigation.

While the intensity of the humoral response may be an indicator of worse outcome, its role in the pathogenesis of severe COVID-19 is not fully elucidated and there are other immunologic features such as the cytokine profiles, inflammatory markers, or lymphocyte defects, which contribute to this pathogenesis [11]. The identification of more complex and specific immunophenotypes may be accurate to predict the risk of severe COVID-19 [11]. Nonetheless, our data suggest that a unique measurement of IgG titers at admission might be a pragmatic stratification tool to identify patients at risk of complications and death. Further analyses in larger datasets including prospective interventional studies could demonstrate whether this approach would be useful to identify patients deserving early additional preventive or therapeutic measures.

## Figures and Tables

**Table 1:** The baseline demographic characteristics and comorbidities of patients.

Characteristics, n (%)	Total (n = 70)	Severe Disease (n = 22)	Moderate Disease (n = 29)	Mild Disease (n = 19)	*p*_Value
Male sex	40 (57)	14 (64)	16 (55)	10 (53)	0.74
Median age, year (IQR)	60 (51–73)	60 (54–64)	60 (51–74)	59 (43–78)	0.80
Obesity (BMI ≥ 30)	25 (36)	8 (36)	15 (52)	2 (11)	**0.01**
Active or prior smoker	12 (17)	2 (9)	8 (28)	2 (11)	0.14
Hypertension	34 (49)	11 (50)	16 (55)	7 (37)	0.45
History of cardiac rhythm or conduction disorder	8 (11)	1 (5)	6 (21)	1 (5)	0.12
Coronary disease	5 (7)	0 (0)	4 (14)	1 (5)	0.40
Heart failure	8 (11)	1 (5)	6 (21)	1 (5)	0.12
Stroke	3 (4)	0 (0)	2 (7)	1 (5)	0.92
Chronic kidney disease (eGFR < 60)	10 (14)	1 (5)	5 (17)	4 (21)	0.27
Chronic pulmonary disease *	10 (14)	3 (14)	6 (21)	1 (5)	0.32
Dyslipidemia	17 (24)	3 (14)	13 (45)	1 (5)	**0.01**
History of thromboembolic event	5 (7)	1 (5)	4 (14)	0 (0)	0.40
Malignancy	8 (11)	2 (9)	2 (7)	4 (21)	0.29
Diabetes	**17 (24)**	**4 (18)**	**10 (34)**	**3 (16)**	**0.24**
Connective tissue disease	2 (3)	0 (0)	2 (7)	0 (0)	0.91
Transplantation (SOT and HSCT)	3 (4)	1 (5)	1 (3)	1 (5)	0.95

Results expressed as total number (percentage) unless otherwise specified. * Includes chronic obstructive pulmonary disease, asthma, and pulmonary fibrosis.

**Table 2:** The clinical characteristics and laboratory values of patients on admission.

Characteristics	Total (n = 70)	Severe Disease (n = 22)	Moderate Disease (n = 29)	Mild Disease (n = 19)	*p*_Value
Median duration of symptoms before admission (IQR)	10 (6–13)	13 (9–15)	8 (7–12)	9 (3–11)	0.55
Fever, n (%)	48 (69)	19 (86)	19 (66)	10 (53)	0.06
Angina, n (%)	7 (10)	2 (9)	4 (14)	1 (5)	0.61
Rhinorrhea, n (%)	5 (7)	1 (5)	3 (10)	1 (5)	0.67
Cough (productive or not), n (%)	49 (70)	18 (82)	22 (76)	9 (47)	**0.03**
Anosmia/dysgeusia, n (%)	9 (13)	1 (5)	6 (21)	2 (11)	0.21
Dyspnea, n (%)	42 (60)	18 (82)	20 (69)	4 (21)	**<0.01**
Diarrhea, n (%)	16 (23)	4 (18)	8 (28)	4 (21)	0.71
Radiological infiltrate, n (%)	49/61 (80)	20/21 (95)	24/27 (89)	5/13 (38)	**<0.01**
White Blood cell count, G/L	7.6 (5.5–9.2)	8 (6.9–10.5)	7.4 (5.2–8.8)	6.9 (5.1–9.1)	0.10
Lymphocytes count, G/L	0.9 (0.7–1.2)	0.9 (0.5–1.1)	0.9 (0.7–1.2)	1.1 (0.7–1.8)	0.40
Platelets count, G/L	216 (161–308)	209 (158–291)	202 (156–280)	244 (212–323)	0.53
D-dimer, ng/ml	1016 (521–1799)	1127 (828–2677)	752 (507–1656)	881 (468–1419)	**0.03**
Creatinine, µmol/L	88 (71–119)	93 (81–105)	88 (77–124)	74 (69–115)	0.66
CRP, mg/L	86 (38–151)	156 (83–273)	88 (53–125)	11 (4–71)	**<0.01**
Procalcitonin, µg/L	0.2 (0.1–0.5)	0.5 (0.2–1.3)	0.2 (0.1–0.3)	0.2 (0.1–0.2)	0.39
Serum ferritin, µg/L	997 (434–1690)	1296 (673–2117)	976 (365–1639)	823 (397–1198)	0.10
Troponin, ng/ml	12 (6–40)	15 (9–125)	10 (6–30)	11 (6–20)	0.06
AST, U/L	57 (33–89)	71 (53–88)	54 (31–92)	36 (25–53)	0.50
ALT, U/L	38 (22–65)	50 (37–69)	37 (20–58)	26 (17–32)	0.49
Bilirubin, µmol/L	9 (7–13)	9 (6–13)	9.5 (6–12)	8 (7–10)	0.60
ABO Group O, n (%)	22/38 (58)	6/16 (38)	8/10 (80)	8/12 (67)	0.25

Results expressed as median values (interquartile range [IQR]), unless otherwise specified.

**Table 3:** Diagnostic performances of the selected tests to discriminate severely ill patients.

Selected Test (Threshold Value)	Sensitivity % (95% CI)	Specificity % (95% CI)	PPV % (95% CI)	NPV % (95% CI)
CLIA (>50.8 UA/mL)	82 (61–93)	79 (65–88)	63 (49–76)	91 (80–96)
Luminex (MFI ratio >77.6)	72 (52–87)	81 (67–90)	64 (48–77)	86 (76–93)
D-dimer (>1000 ng/mL)	71 (48–89)	59 (42–75)	44 (33–56)	82 (69–91)
CRP (>100 mg/L)	68 (45–86)	73 (57–85)	56 (42–69)	82 (71–90)

95% CI = 95% confidence interval, MFI = median fluorescence intensity, PPV = positive predictive value, NPV = negative predictive value.

## Data Availability

Data supporting the reported results are available upon request for the peer-review process.

## Supplementary Materials

The following supporting information can be downloaded at: www.mdpi.com/article/10.3390/v14051089/s1, Figure S1: Antibody titers stratified by duration of symptoms; Table S1: Administered anti-SARS-CoV-2 treatment.
