# Supplementary material for: Anti-SARS-CoV-2 Titers Predict the Severity of COVID-19
Source: Viruses. 2022 May 18;14(5):1089. doi: 10.3390/v14051089 (PMC9143418; doi:10.3390/v14051089)
Supplement: Supplementary file 1 [file viruses-14-01089-s001.zip › viruses-1690908-supplementary.pdf]

Supplementary Material

Anti-SARS-CoV-2 Titers Predict the Severity of COVID-19

A. Kritikos[1,2^], S. Gabellon[2^], JL. Pagani[3], M. Monti[4], PY. Bochud[2], O. Manuel[2], A. Coste[1], G. Greub[1,2], M. Perreau[5], G. Pantaleo[5], A. Croxatto[1,6], F. Lamoth[1,2]

[1] Institute of Microbiology, Lausanne University Hospital and University of Lausanne; antonios.kritikos@chuv.ch (AK); Alix.Coste@chuv.ch (ACO); Antony.Croxatto@ne.ch (AC); Gilbert.Greub@chuv.ch (GG); Frederic.Lamoth@chuv.ch (FL)

[2] Infectious Diseases Service, Lausanne University Hospital and University of Lausanne; sophie.gabellon@unil.ch (SG); Pierre-Yves.Bochud@chuv.ch (PYB), Oriol.Manuel@chuv.ch (OM)

[3] Intensive Care Unit, Lausanne University Hospital and University of Lausanne; Jean-Luc.Pagani@chuv.ch (JLP)

[4] Service of Internal Medicine, Lausanne University Hospital and University of Lausanne; Matteo.Monti@chuv.ch (MM)

[5] Service of Immunology and Allergy, Lausanne University Hospital and University of Lausanne; Matthieu.Perreau@chuv.ch (MP); Giuseppe.Pantaleo@chuv.ch (GP)

[6] ADMED Microbiology, La Chaux-de-Fonds, Switzerland

* Correspondence: Antonios.kritikos@chuv.ch; Tel: +41-(0)-21-314-02-71

^ Equal contribution

Figure S1. Antibody titers stratified by duration of symptoms.

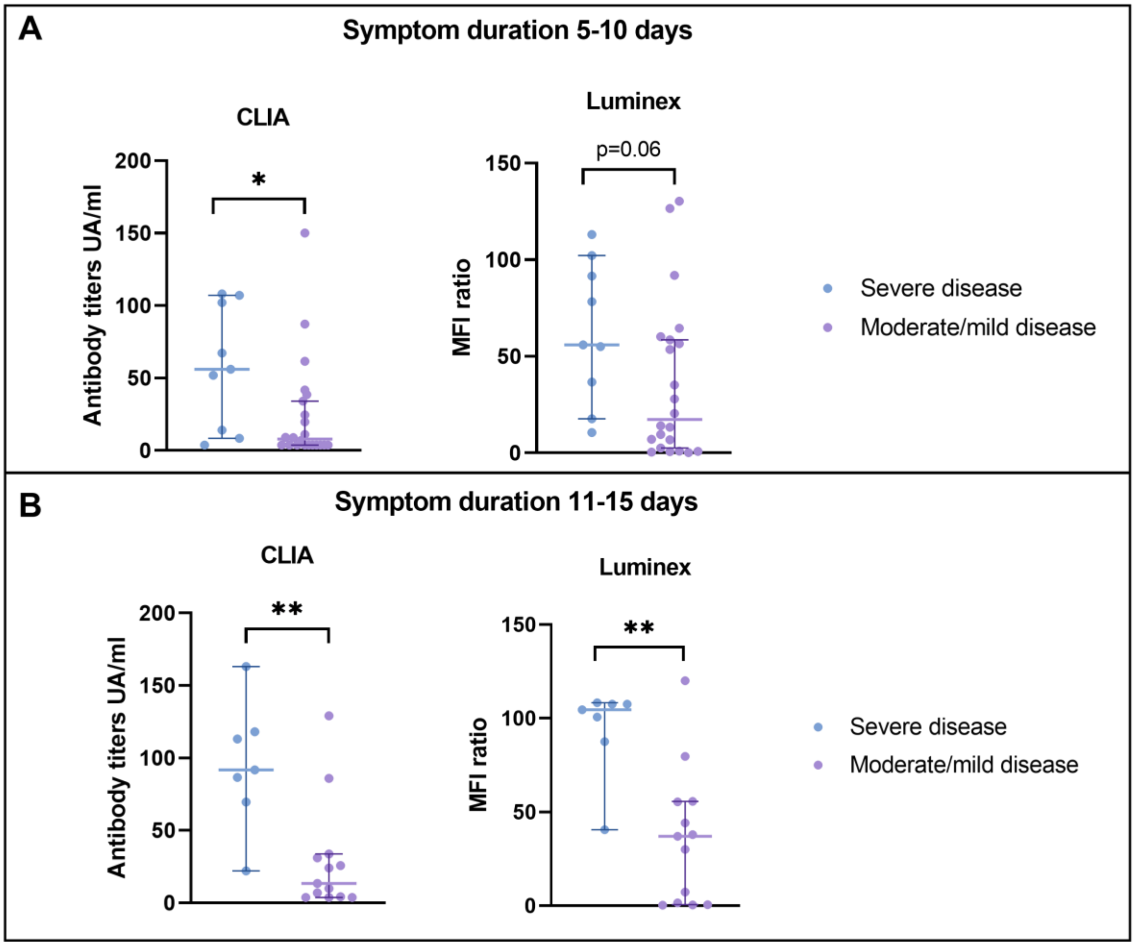

Scatter plot with lines at median and whiskers showing the 95% confidence interval (CI). **Panel A)** Symptom duration 5-10 days**, panel B)** symptom duration 11-15 days.(*=p<0.05, **=p<0.01, MFI= Median fluorescence intensity).

Table S1: Administered anti-SARS-CoV-2 treatment.

Characteristics, n (%)	Total patients (N = 70)	Severe disease	Moderate disease	Mild disease	p_value
Any anti-SARS-CoV-2 treatment	44 (63)	22 (31)	20 (29)	2 (3)	**<0.001**
Protease inhibitor[1]	14 (32)	6 (27)	7 (35)	1 (50)	0.3
Hydroxychloroquine	27 (61)	12 (54)	13 (65)	2 (100)	**0.01**
Remdesivir	8 (18)	6 (27)	2 (10)	0	**<0.001**
Tocilizumab	20 (45)	15 (68)	5 (25)	0	**<0.01**
Corticosteroid treatment	7 (16)	5 (23)	2 (10)	0	0.1
Interferon-based therapy	2 (4)	0	2 (10)	0	0.9

[1] Lopinavir or atazanavir
